# Supplementary material for: Oxygen Vacancy-Mediated Activates Oxygen to Produce Reactive Oxygen Species (ROS) on Ce-Modified Activated Clay for Degradation of Organic Compounds without Hydrogen Peroxide in Strong Acid
Source: Nanomaterials (Basel). 2022 Dec 10;12(24):4410. doi: 10.3390/nano12244410 (PMC9785360; doi:10.3390/nano12244410)
Supplement: Supplementary file 1 [file nanomaterials-12-04410-s001.zip › nanomaterials-2013376-supplementary.pdf]

nanomaterials

Supplementary Material

Oxygen Vacancy-Mediated Activates Oxygen to Produce Reactive Oxygen Species (ROS) on Ce-Modified Activated Clay for Degradation of Organic Compounds without Hydrogen Peroxide in Strong Acid

Tianming Wu [1], Jing Cui [2], Changjiang Wang [3], Gong Zhang [4], Limin Li [1], Yue Qu [1] and Yusheng Niu [1,5,*]

1 Institute of Biomedical Engineering, College of Life Sciences, Qingdao University, Qingdao 266071, China
2 College of Resources and Environment, Shandong Agricultural University, Taian 271018, China
3 Shandong Zhengyuan Geological Resource Exploration Co. Ltd., China Metallurgical Geology Bureau, Weifang 261200, China
4 Center for Water and Ecology, State Key Joint Laboratory of Environment Simulation and Pollution Control, School of Environment, Tsinghua University, Beijing 100084, China
5 School of Tourism and Geography Science, Qingdao University, Qingdao 266071, China
* Correspondence: nys@qdu.edu.cn; Tel.: +86-532-85955399

Supplementary Figures

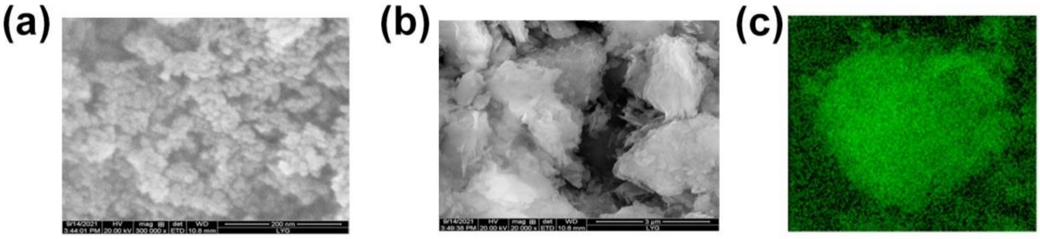

Figure S1. SEM images of CeO_2（**a**）， Acid treated clay (**b**). EDS compositional mapping of O (**c**).

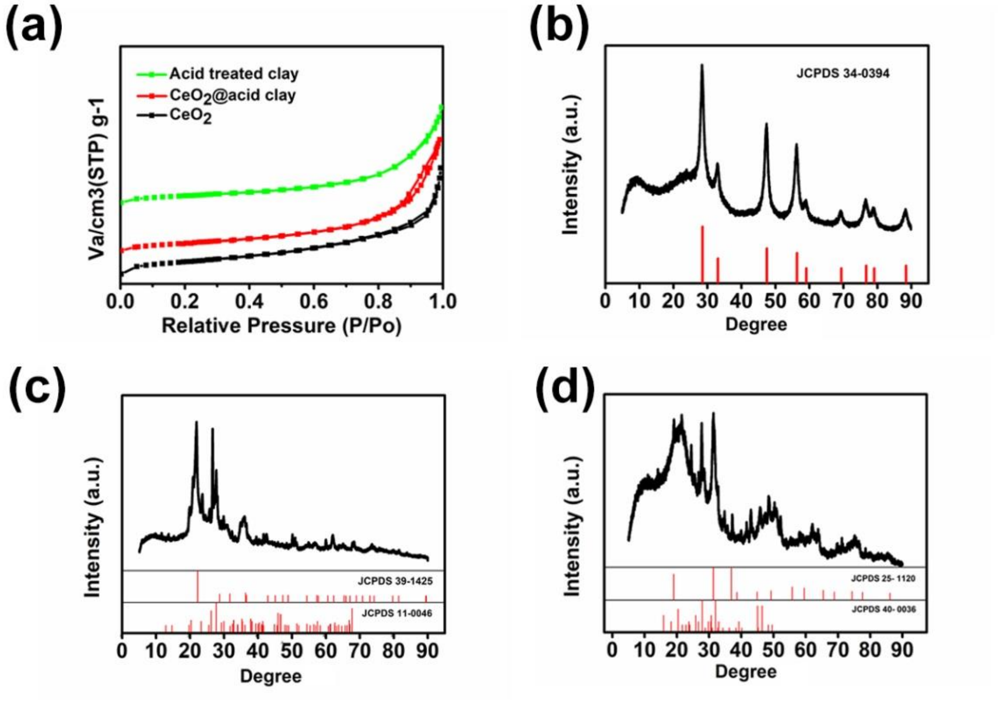

Figure S2. N_2 adsorption/desorption isotherms of CeO_2, acid treated clay and CeO_2@acid clay (**a**). XRD patterns of the CeO_2 (**b**), Acid treated clay (**c**) and CeAY(**d**).

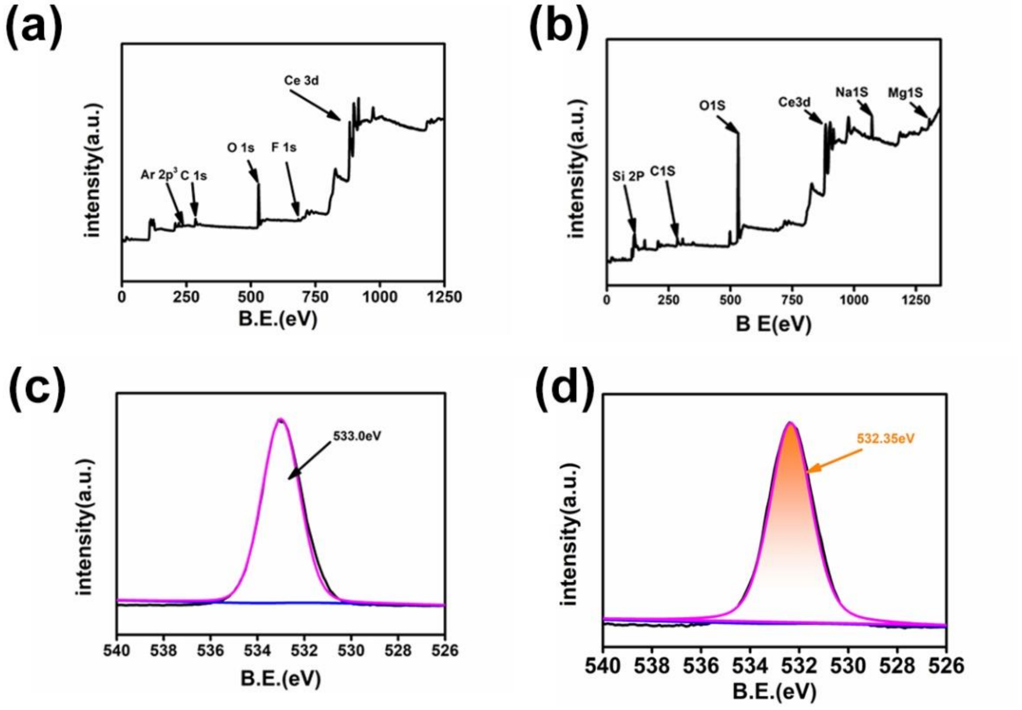

Figure S3. The XPS survey spectrum of CeO_2 (**a**) and CeO_2@acid clay (**b**). The O1s of Acid treated clay (**c**). The O1S XPS patterns of CeAY after degradation of MO (**d**).

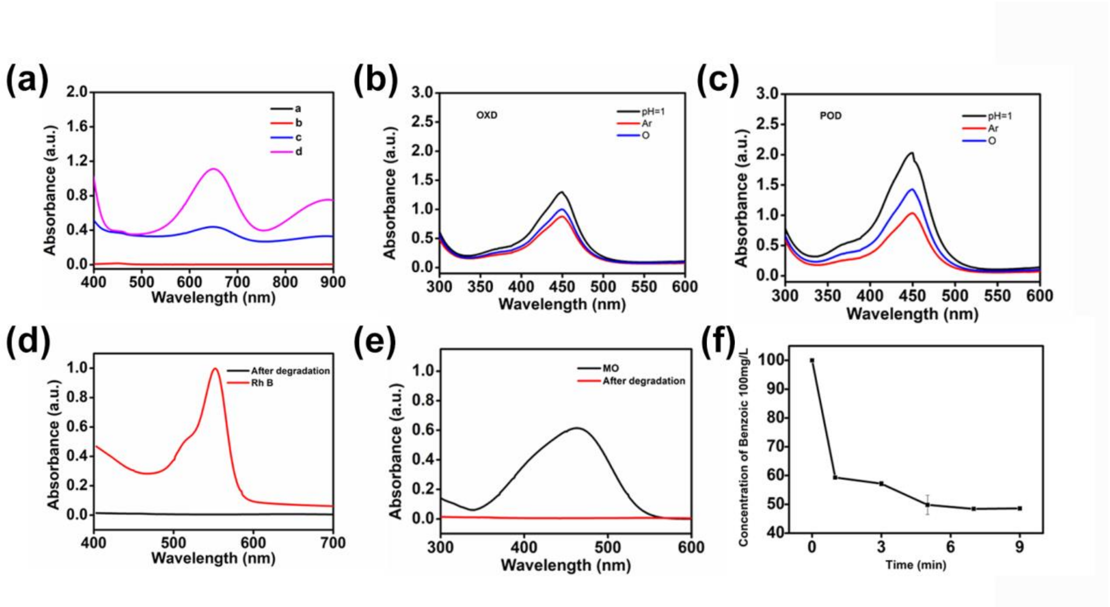

Figure S4. The catalyze activity of the CeO_2, a)TMB; b)TMB + H_2O_2; c)CeO_2 + TMB; d)CeO_2 + TMB + H_2O_2 (**a**). The effort of the oxygen, argon to the catalyze activity of CeAY (**b**,**c**). The degradation result of Rh B, MO and Benzoic acid (CeAY 2 mg mL^{-1}, room temperature pH = 1, 5 mg L^{-1}) (**d**–**f**).

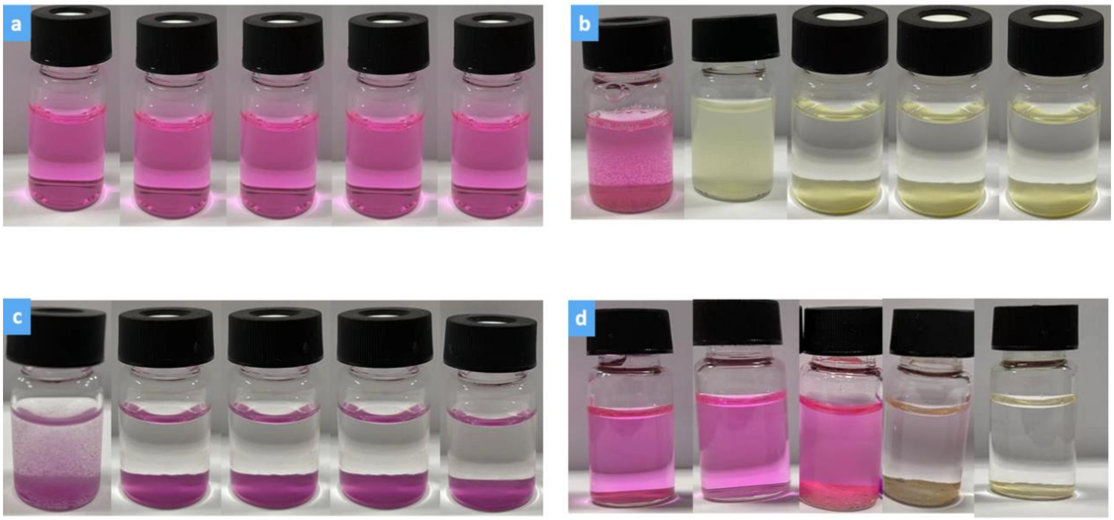

Figure S5. The degradation effect of the Rh B with time increased (a, pH = 7.0, after adding the CeO_2 and CeAY; b, pH = 1.0, after adding the CeAY; c, the acid-treated clay adsorbs Rh B to the bottom of the solution; d, pH = 1.0, after adding the CeO_2 for two weeks).

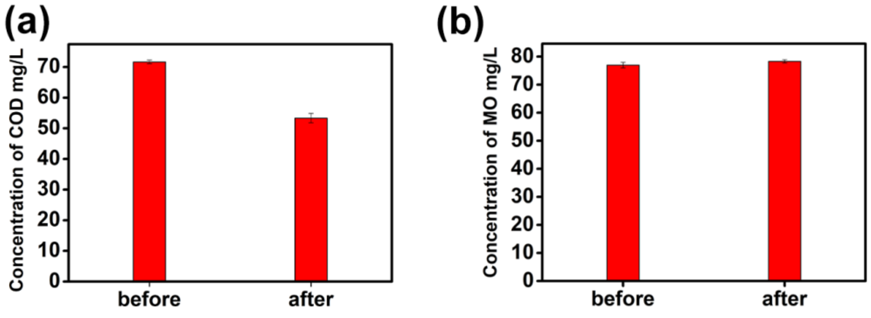

Figure S6. The Concentration of COD before and after degradation (a, Rh B; b, MO).

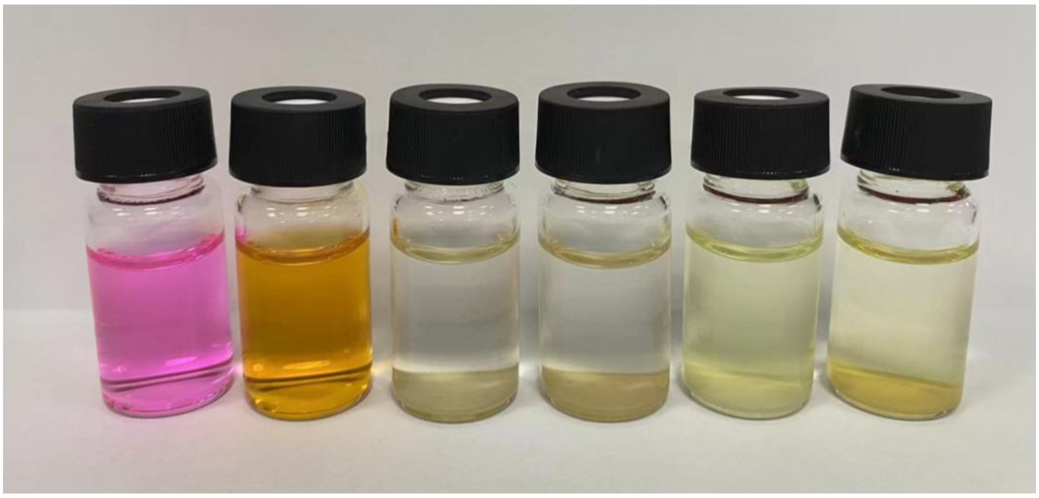

Figure S7. The degradation effect of Rb B and MO at different pH and different strong acid.

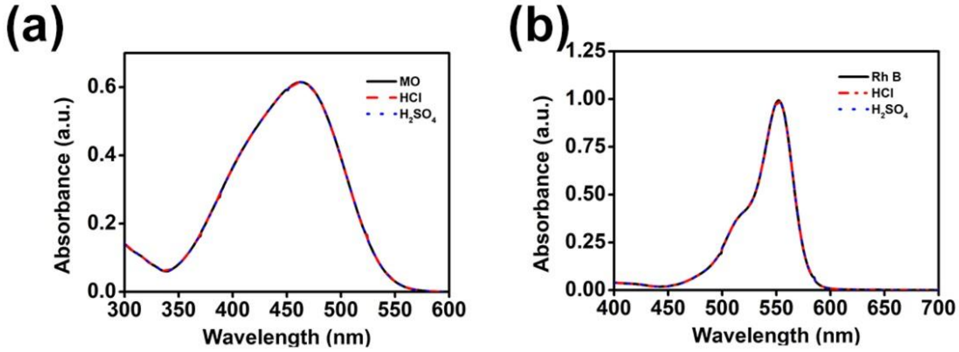

Figure S8. The effect of add HCl and H_2SO_4 on the degradation of Mo and Rh B (a, MO; b, Rh B).

Supplementary Tables

Table S1. The main components of bentonite.

component	SiO_2	Al_2O_3	Fe_2O_3	FeO	TiO	CaO	MgO	MnO	K_2O	Na_2O	P_2O_5
content	61.74	16.08	3.19	0.27	0.15	5.01	3.19	0.29	1.00	0.22	0.03

Table S2. The specific surface area of different materials.

Sample	Surface area(m^2g^{-1})
CeO_2	2.6143
Acid treated clay	68.641
CeO_2@acid clay	94.463

Table S3. The Michaelis-Menten constant (K_m) and maximum reaction rate (V_{max}) of the as-obtained CeAY with TMB as the substrate.

Catalyst	Substance	K_m (mM)	V_{max}(10^{-7}M/s)	References
CeAY	TMB	0.3412	0.0127	This work
CeO_2	TMB	1.5	0.69	[1]
isPNC	TMB	3.8	7	[2]
swPNC	TMB	1.9	6	[2]
isDNC	TMB	1.8	5	[2]
swDNC	TMB	0.8	3	[2]
HRP	TMB	0.43	1	[3]
Cu-Cys@COF-OMe	TMB	0.75	1.01	[4]
CH-Cu	TMB	0.58	4.56	[5]

Table S4. Degradation intermediates of RhB in the CeAY system.

Relative molecular mass	Structure
165	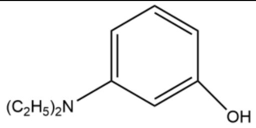
148	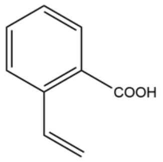
230	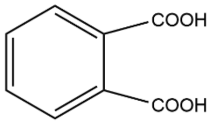
110	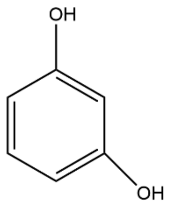
218	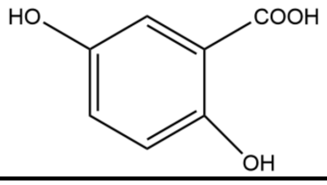

154	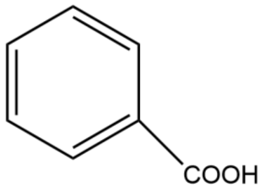
196	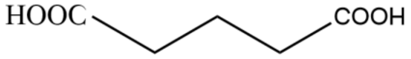
168	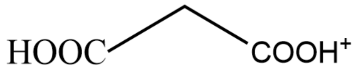
154	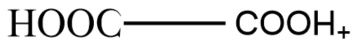
46	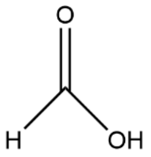

Table S5. Degradation intermediates of RhB in the CeAY system.

Relative molecular mass	Structure
304	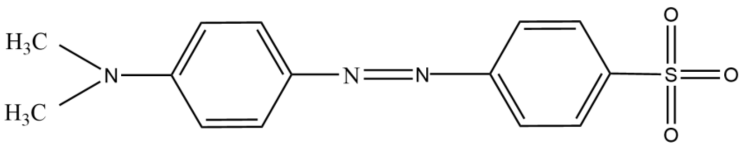
306	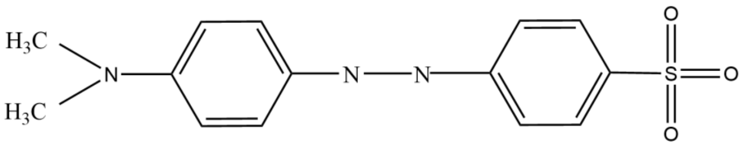
184	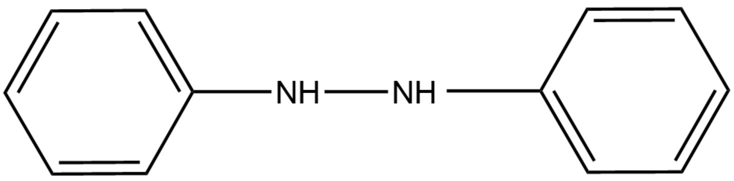
157	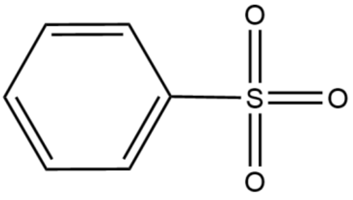
142	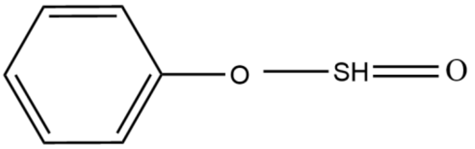

121	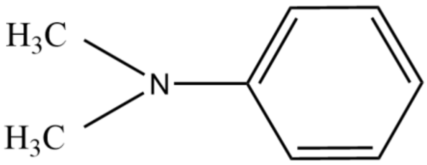
108	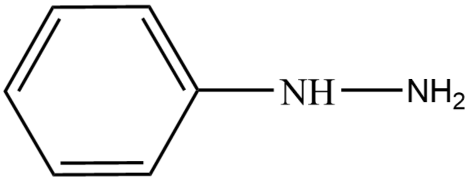
107	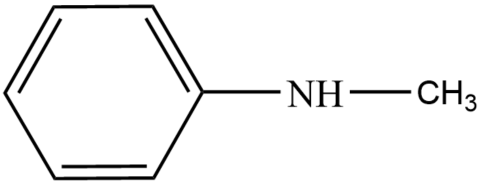
102	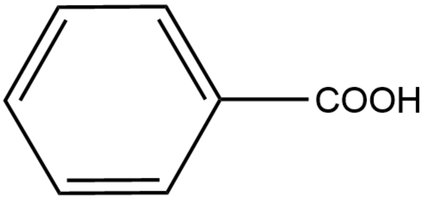
82	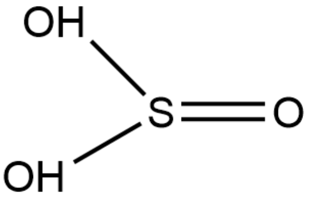

[1] B. Liu, Z. Huang, J. Liu, Boosting the oxidase mimicking activity of nanoceria by fluoride capping: rivaling protein enzymes and ultrasensitive F(-) detection, Nanoscale, 8 (2016) 13562-13567.

[2] A. Asati, S. Santra, C. Kaittanis, S. Nath, J.M. Perez, Oxidase-like activity of polymer-coated cerium oxide nanoparticles, Angew Chem Int Ed Engl, 48 (2009) 2308-2312.

[3] L. Gao, J. Zhuang, L. Nie, J. Zhang, Y. Zhang, N. Gu, T. Wang, J. Feng, D. Yang, S. Perrett, X. Yan, Intrinsic peroxidase-like activity of ferromagnetic nanoparticles, Nature Nanotechnology, 2 (2007) 577-583.

[4] Y. Tang, S. Jiang, W. Li, S. Jalil Shah, Z. Zhao, L. Pan, Z. Zhao, Confined construction of COF@Cu-nanozyme with high activity and stability as laccase biomimetic catalyst for the efficient degradation of phenolic pollutants, Chemical Engineering Journal, 448 (2022).

[5] J. Wang, R. Huang, W. Qi, R. Su, B.P. Binks, Z. He, Construction of a bioinspired laccase-mimicking nanozyme for the degradation and detection of phenolic pollutants, Applied Catalysis B: Environmental, 254 (2019) 452-462.
